# T4 + T3 combination therapy: any progress?

**DOI:** 10.1007/s12020-019-02052-2

**Published:** 2019-10-15

**Authors:** Wilmar M. Wiersinga

**Affiliations:** Department of Endocrinology & Metabolism, Academic Medical Center, University of Amsterdam, Amsterdam, The Netherlands

**Keywords:** Hypothyroidism, Combination therapy, T4, T3

## Abstract

Guidelines on T4 + T3 combination therapy were published in 2012. This review investigates whether the issue is better understood 7 years later. Dissatisfaction with the outcome of T4 monotherapy remains high. Persistent symptoms consist mostly of fatigue, weight gain, problems with memory and thinking and mood disturbances. T4 monotherapy is associated with low serum T3 levels, which often require TSH-suppressive doses of L-T4 for normalization. Peripheral tissue thyroid function tests during T4 treatment indicate mild hyperthyroidism at TSH < 0.03 mU/L and mild hypothyroidism at TSH 0.3–5.0 mU/L; tissues are closest to euthyroidism at TSH 0.03–0.3 mU/L. This is explained by the finding that whereas T4 is usually ubiquinated and targeted for proteasomal degradation, hypothalamic T4 is rather stable and less sensitive to ubiquination. A normal serum TSH consequently does not necessarily indicate a euthyroid state. Persistent symptoms in L-T4 treated patients despite a normal serum TSH remain incompletely understood. One hypothesis is that a SNP (Thr92Ala) in *DIO2* (required for local production of T3 out of T4) interferes with its kinetics and/or action, resulting in a local hypothyroid state in the brain. Effective treatment of persistent symptoms has not yet realized. One may try T4 + T3 combination treatment in selected patients as an experimental *n* = 1 study. The 2012 ETA guidelines are still valid for this purpose. More well-designed randomized clinical trials in selected patients are key in order to make progress. In the meantime the whole issue has become rather complicated by commercial and political overtones, as evident from skyrocketing prices of T3 tablets, aggressive pressure groups and motions in the House of Lords.

## Introduction

In 2012 the European Thyroid Association (ETA) published the first guidelines on T4 + T3 combination therapy in hypothyroidism [1]. It is now 7 years later, and one wonders if any progress has been made. I would like to address this issue by asking the same questions as in the 2012 guidelines.

## Is there an unmet need in L-T4 treated hypothyroid patients?

In 2012 it was reported that 5–10% of L-T4 treated hypothyroid patients with normal serum TSH have persistent symptoms (impaired well-being, psychological distress and cognitive disturbances) that can be related to the thyroid disease and L-T4 therapy [1]. This statement was based on three population-based studies from the UK, the Netherlands and Norway [2–4]. Since then there have been no more studies in the general population, but in 2017 an online survey for hypothyroid patients posted on the American Thyroid Association (ATA) website demonstrates prominent dissatisfaction with treatment [5]. Among respondents without self-reported depression, life stressors, or medical conditions (*n* = 3670), satisfaction on a scale of 1–10 with L-T4 was 5 (IQR 3–7), satisfaction with L-T4 + L-T3 was 6 (IQR 3–8), and satisfaction in patients taking desiccated thyroid extract (DTE) was highest (score 7, IQR 5–9) (*p* < 0.0001). Among those who were frustrated with their treatment (~22% of respondents taking L-T4 or L-T4 + L-T3, and 14% of DTE users), the relevant areas identified as causing dissatisfaction were fatigue or energy level (77%), weight management (69%), memory or other problems with thinking (58%), and mood (45%) [5]. The survey results are probably biased as dissatisfied patients are more likely to have participated in the survey than patients who felt happy with their treatment. Another bias might be a relatively high proportion of subclinical hypothyroidism among the participants, since thyroxine replacement in the last decades is initiated at progressively lower serum TSH concentrations (in 2009 at a median TSH of 7.9 mU/L) [6]. Recent trials have not shown improvement in thyroid-related symptoms or quality of life after levothyroxine treatment of subclinical hypothyroidism, especially not in older individuals [7–9]. Dissatisfaction with treatment outcome could thus be related to inclusion of many older patients with subclinical hypothyroidism.

## Is there a biologic rationale for persistent complaints in L-T4 treated hypothyroid patients?

In 2012 five possible causes of persistent complaints were identified [1].

### 1. Nonspecific causes related to the chronic nature of the disease

Awareness of having a chronic disease and lifelong dependency of thyroid medication could make patients unhappy and less healthy [1]. There have been no prospective studies to test the hypothesis that attitudes, health perceptions and mood of patients prior to L-T4 treatment differ between those with and without persistent complaints after treatment. Qualitative interview studies show that patients in general have a low understanding of their condition [10]. When they experience hypothyroid symptoms at initial diagnosis, the perception of disease susceptibility (and adherence to L-T4) is better, but patients who remain unwell despite a normal serum TSH felt that their normal result presented a barrier to further evaluation. Qualitative studies among general practitioners and nurses reveal inadequate knowledge of medication interactions and L-T4 pharmacokinetics [11].They rely on blood tests over clinical symptoms to adjust L-T4 dose. Information exchange is usually restricted by time and often centered on symptoms rather than patient education. Poor adherence was felt to be the main reason for suboptimal treatment, although other factors such as comorbidity and comedication were mentioned. It follows that improvement of the interaction between physicians and patients could reduce barriers to optimal thyroid replacement.

### 2. Associated autoimmune diseases

Associated autoimmune diseases occur in 14% of patients with Hashimoto’s disease and in 10% of patients with Graves’ disease [12]. It is unknown how often overlooked associated autoimmune diseases are responsible for persistent complaints.

### 3. Thyroid autoimmunity per se

Whether or not thyroid autoimmunity per se might be responsible for particular symptoms, remains unclear. For instance, the mere presence of TPO antibodies in otherwise euthyroid subjects has been linked to depression in some population-based studies but not in others [1]. Complete removal of thyroid antigens by total thyroidectomy induces progressive disappearance of thyroid antibodies. To determine whether thyroidectomy improves symptoms which persisted in patients with Hashimoto thyroiditis despite having normal thyroid function tests while receiving thyroid hormone replacement therapy, a randomized clinical trial was done comparing total thyroidectomy with continuation of medical therapy [13]. Total thyroidectomy improved health-related quality-of-life and fatigue, whereas medical therapy did not. At 18 months, median TPO-Ab values were reduced from 2232 to 152 kU/L in the surgical group and from 2052 to 1300 kU/L in the medical group.

### 4. Inadequacy of L-T4 dose

In accordance with previous studies, NHANES participants using L-T4 and having a normal serum TSH, experience higher total and free T4 and lower total and free T3 serum concentrations than healthy or matched controls [14]. FT3 values below the lower normal limit are observed in about 15% of hypothyroid patients on L-T4 [15]. One may thus question whether low serum T3 levels could be involved in persistent complaints, necessitating higher L-T4 doses to normalize T3 levels. This seems, however, not very likely. Slightly lower or higher L-T4 doses did not produce measurable changes in hypothyroid symptoms, well-being or quality of life [16, 17], nor in substantial metabolic differences [18]. Another study evaluated the effect of total thyroidectomy, which abolishes thyroidal T3 secretion [19]. Postoperative FT3 levels during L-T4 replacement were compared with preoperative native serum FT3 in the same individuals: postoperative FT3 was higher than preoperative FT3 in subjects with postoperative TSH <0.03 mU/L, unchanged in subjects with postoperative TSH 0.03–0.3 mU/L, and lower in subjects with postoperative TSH 0.3–5.0 mU/L [19]. These studies suggest that TSH-suppressive doses of L-T4 are required to achieve serum T3 levels similar to those before thyroidectomy. It provides a rationale for T3 supplementation [20]. However, there is no good evidence that low serum T3 or low FT3/FT4 ratios are linked to persistent symptoms. The mechanism responsible for the low serum T3 during L-T4 therapy has to do with type 2 iodothyronine deiodinase (DIO2). This enzyme catalyzes the deiodination of T4 into T3 in extrathyroidal tissues thereby sustaining serum T3 levels during L-T4 replacement. DIO2 has a short half-life (≈60 min.) that becomes even shorter (20 min.) by interacting with T4, which results in DIO2 ubiquitination and targeting for proteosomal degradation [21]. DIO2 in the hypothalamus, in contrast to other tissues, is rather stable and less sensitive to ubiquitination [22]. Thus, whereas in the rest of the body DIO2-mediated T3 production progressively decreases with increasing L-T4 doses because of DIO2 ubiquitination, T3 production in the hypothalamus/pituitary is not and the dose of L-T4 required to normalize serum TSH is lower than the dose that normalizes serum T3. It follows that in order to reach a normal serum T3, one has to administer a relatively high L-T4 dose that is likely to suppress serum TSH.

### 5. Inadequacy of L-T4 treatment modality

Peripheral tissue thyroid function tests have been evaluated before total thyroidectomy and at 1 year postoperatively when using L-T4 [23]. Patients who had postoperative TSH ≤ 0.03 mU/L, were mildly hyperthyroid at tissue level, those with TSH between 0.03 and 0.3 mU/L had peripheral tissue function tests closest to euthyroidism, and those with TSH between 0.3 and 5.0mU/L had mild tissue hypothyroidism (Table 1). L-T4 replacement in doses that normalize serum TSH, do not normalize all systemic markers of thyroid hormone signaling, including serum LDL cholesterol and total cholesterol [24]. A normal serum TSH is thus no guarantee for a euthyroid state in all target tissues [15, 25]. One must conclude that L-T4 therapy is incapable to achieve euthyroidism simultaneously in all target tissues, precisely the conclusion reached by the Escobar’s in their now famous experiments on thyroid hormone replacement in hypothyroid rats: only the combination of L-T4 + L-T3 could ensure simultaneous euthyroidism in all tissues [1]. Although L-T4 may not be the ideal form of thyroid hormone replacement, the vast majority of patients are satisfied with its outcome. Why a subset of patients keeps persistent symptoms, remains incompletely understood. It has been hypothesized that a particular single nucleotide polymorphism (SNP) in *DIO2* is involved, namely Thr92Ala. Interest in this SNP was raised by the early finding that Thr92Ala was associated with impaired psychological well-being on L-T4 therapy and enhanced response to T4 + T3 combination therapy [26]. Up to 80% of intracellular T3 in brain is derived from local deiodination of T4 into T3 catalyzed by D2. Some studies demonstrate reduced D2 activity in the presence of Thr92Ala [27], but others observe normal enzyme kinetics of the SNP [28]. A Dutch population-based study reports that the Ala/Ala genotype of this D2 polymorphism is present in 11.3% of T4 users and in 10.7% of the general population; in both groups the SNP is associated neither with differences in serum TSH, FT4, FT3, or FT3/FT4 ratio, nor with health-related quality of life and cognitive functioning [29]. Recently the cellular abnormalities associated with the Thr92Ala protein have been explored further. The Ala92 version of the protein has a longer half-life than the wild type, is ectopically localized in the Golgi apparatus, and alters the genetic profile of certain areas of the human brain in a pattern reminiscent of neurodegenerative disease, without evidence of reduced thyroid hormone signaling [30]. The latest study reports D2 is a cargo protein, recycling between ER and Golgi [31]. The Thr92-to-Ala substitution causes ER stress, activates the unfolded protein response (UPR), accumulates in the trans-Golgi, and generates less T3. Mouse carrying Ala92 *DIO2* exhibit UPR and hypothyroidism in distinct brain areas, whereas exogenous L-T3 improves cognition. Primary hypothyroidism intensifies the Ala92 *DIO2* phenotype, with only partial response to L-T4. One has to conclude that the origin of persistent complaints in L-T4 treated hypothyroid patients who have a normal serum TSH, is still incompletely understood. On the other hand, one can also conclude that L-T4 monotherapy is unlikely to be the ideal mode of thyroid hormone replacement. A 2013 survey among endocrinologists indicated that persistent symptoms despite achieving target TSH values, would prompt testing for other causes by 84% of respondents, a referral to primary care by 11%, and a change to L-T4 + L-T3 combination therapy by 3.6%; 22% would ask for measurement of T3 [32].

**Table 1:** Peripheral tissue thyroid function tests in 133 patients before total thyroidectomy and at one year postoperatively under L-T4 medication [23]

Postoperative TSH	≤0.03 mU/L (*n* = 58)	0.03 to ≤0.3 mU/L (*n* = 46)	0.3 to ≤5.0 mU/L (*n* = 29)
	Preop → postop	Preop → postop	Preop → postop
TSH mU/L	1.48 → 0.01 ↓	1.56 → 0.07 ↓	1.59 → 1.51 NS
FT4 ng/dl	1.07 → 1.56 ↑	1.09 → 1.45 ↑	1.12 → 1.38 ↑
FT3 pg/ml	2.79 → 3.17 ↑	2.92 → 2.96 NS	2.92 → 2.76 ↓
*Thyroid state in serum postop*.	*→* *Hyperthyroid*	*→* *Subclinical hyperthyroid*	*→* *Euthyroid*
LDL-C mg/dl	114 → 111 NS	104 → 104 NS	108 → 114 ↑
SHBG nmol/l	69 → 82 ↑	66 → 66 NS	67 → 72 NS
TRACP mU/dl	377 → 371 NS	361 → 328 NS	362 → 319 ↓
BAP μg/dl	13 → 15 ↑	13 → 13 NS	15 → 14 NS
*Thyroid state in tissues postop*.	*→* *Mild hyperthyroid*	*→* *Closest to euthyroid*	*→* *Mild hypothyroid*

*LDL-C* LDL-cholesterol, *SHBG* sex hormone binding globulin, *TRACP* tartrate-resistant acid phosphatase, *BAP* bone alkaline phosphatase, *NS* not significant, ↓ significant fall, ↑ significant rise

## Is there evidence that L-T4 + L-T3 combination therapy serves the hypothyroid patient better than L-T4 monotherapy?

A 2006 meta-analysis of 11 RCTs comparing L-T4 monotherapy with L-T4 + L-T3 combination therapy found no differences in various outcome measures (quality of life, cognition, mood or symptoms) [1]. Adverse events also did not differ between both regimens. The most recent RCT likewise finds no differences [33]. Many if not all RCTs can be criticized on a number of issues, e.g. selection bias due to inclusion of heterogeneous patient groups by etiology and prognosis, dilution of the true effect by low proportion of symptomatic patients, small sample size, misguided TSH targets, confounding caused by variation in T4 to T3 conversion efficiency, wide variation in treatment response, small effect size on the QoL instrument [34]. In seven of the RCTs patients were asked about their preference for a specific treatment period: 48% preferred T4 + T3 therapy, 25% preferred T4 therapy, and 27% had no preference [1]. Patients randomized to receive T4 + T3 lost 0.5–1.5 kg whereas those randomized to T4 gained 0.1–0.5 kg. Recent studies, however, could not confirm a relationship between preferences and changes in body weight [35, 36]. All guidelines state L-T4 should remain the treatment of choice for hypothyroid patients [37].

## Could it be that trials comparing L-T4 + L-T3 combination therapy and L-T4 monotherapy have not targeted the right population?

This is very well possible. Outcomes might be different by applying different selection criteria. New RCTs can be envisaged restricted to patients with persistent symptoms and/or specific genotypes like Thr92Ala *DIO2*. Other polymorphisms should be considered as well, like SNPs in the brain-specific thyroid hormone transporter *OATP1C1* which have been associated with fatigue and depression but not with neurocognitive functioning or preference for T4 + T3 [1]. Interestingly, a Danish RCT found two SNPs (Thr92Ala *DIO2* and rs17606253 *MCT10* -monocarboxylate transporter 10) associated with favored treatment: preference for T4 + T3 therapy was 42% when both SNPs were absent, 63% if one SNP was present, and 100% if both SNPs were present [35].

## Which patients would qualify for L-T4 + L-T3 combination therapy?

**Fig. 1:**
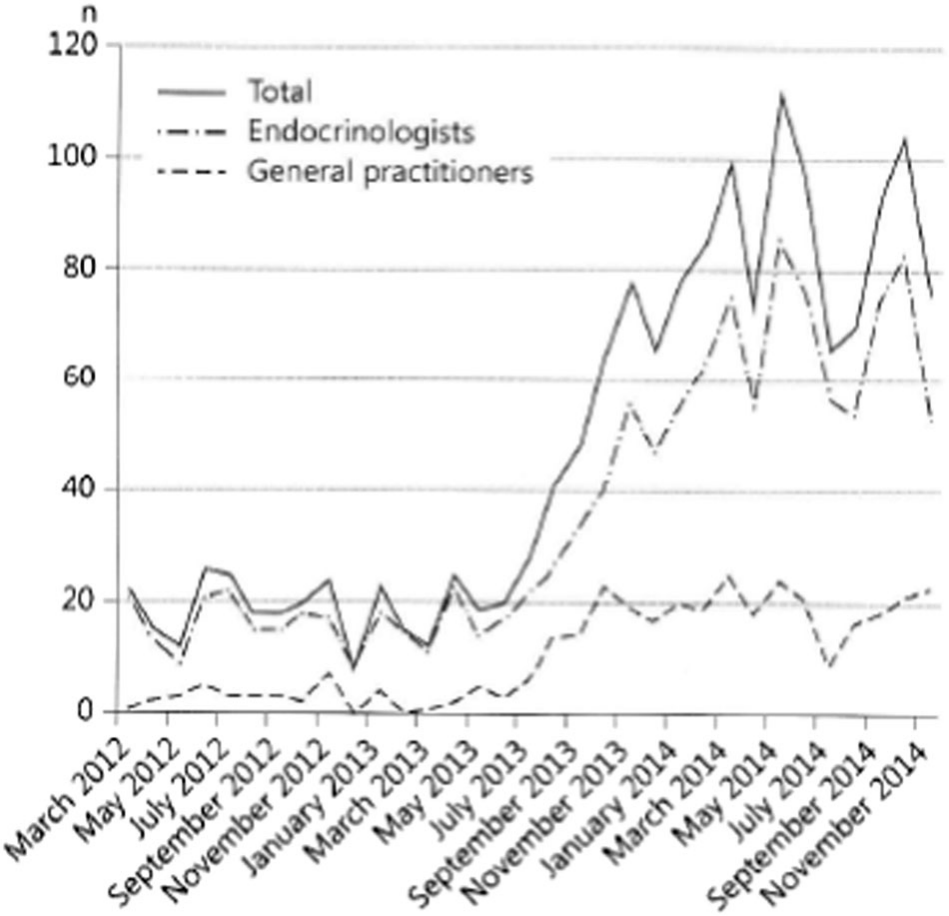
Number of applications for reimbursement of L-T4 + L-T3 combination therapy in Denmark increased 3.8 fold in the period July 2013-June 2014 compared to July 2012–June 2013, most likely provoked by intense media coverage of hypothyroidism and its treatment [41]

The 2012 ETA guidelines suggest “that L-T4 + L-T3 combination therapy might be considered as an experimental approach in compliant L-T4 treated hypothyroid patients who have persistent complaints despite serum TSH values within the reference range, provided they have previously given support to deal with the chronic nature of their disease and associated autoimmune diseases have been ruled out. T4 + T3 combination therapy is not recommended in pregnant women and in patients with cardiac arrhythmias” [1]. These recommendations have been adopted by Italian and British Thyroid Associations, whereas the ATA takes a more neutral position [37]. It is further suggested that the combination therapy is discontinued if no improvement is experienced after three months. Concern about the long-term safety of T4 + T3 combination therapy still exists, but the results of a 17-year observational population-based study in Scotland on liothyronine (T3) use are reassuring [38]. Compared to patients only taking L-T4 (*n* = 33955), those using L-T3 (with or without L-T4, *n* = 400) had no increased risk of cardiovascular disease, atrial fibrillation or fractures after adjusting for age. There was no difference in the number of prescriptions for bisphosphonates or statins, but there was an increased risk of new prescriptions for antipsychotic medication (hazard ratio 2.26, CI 1.64–3.11) which was proportional to the number of L-T3 prescriptions [38]. A 2017 survey among ATA members looked for characteristics that would lead to alternative therapies in T4-treated hypothyroid patients [39]. Especially the presence of symptoms (adjusted odds ratio 25.6), but also low serum T3, the presence of *DIO2* polymorphism and patient request (adjusted odds ratio’s 2.3–2.6) increased physician’s willingness to prescribe T3-containing therapy, whereas older age and comorbidities decreased willingness; athyreotic state, sex and body mass index had no effect. Demands for combination therapy increase in the USA, Canada, Australia, and most European countries [40, 41]. This is well illustrated by a sharp 3.8 fold increase in the number of applications for reimbursement of T4 + T3 therapy in Denmark between the periods July 2012-June 2013 and July 2013-June 2014 (Fig. 1) [41]. This huge increase most likely was caused by extensive media coverage of hypothyroidism and its treatment. It looks the T4 + T3 issue has become a real “hype”. The Danish paper also provides much information on what is going on in real life with respect to combination therapy (Table 2) [41]. Certainly the 2012 ETA guidelines are not followed in many cases. For, the guidelines recommend prescriptions and dose adjustments should be done by accredited internists/endocrinologists and not by general practitioners and not at all by patients themselves. Of concern is the large proportion of patients whose serum TSH at diagnosis was <10 mU/l, meaning they had subclinical hypothyroidism which may lead to less benefit from therapy and possibly overtreatment [7, 8, 42].

**Table 2:** “Real-life” data on T4 + T3 combination therapy in Denmark, according to an internet-based questionnaire study [41]

Respondents	*n* = 293 female 94%, male 6%;<40 years 20%, 41–60 years 63%, ≥ 61 years 17%; 1°/2° education 11%, vocational or short 3° education 28%, 3° education>3 yr 60%, no answer 1%
Duration of hypothyroidism	<1 years 4%, 1–3 years 15%, 3–10 years 36%, >10 years 45%
TSH at diagnosis	Do not remember 26%; < 4 mU/L 26%; 4–10 mU/L 18%; 10–20 mU/L 11%; 20–50 mU/L 6%; >50 mU/L 12%
Symptoms before start of T4 + T3 therapy	Tired 91%, lack of energy 87%, cognitive problems 83%, musculoskeletal symptoms 76%, weight problems 75%, pain 49%, constipation 42%, depression 39%
Prescriptions	L-T4 + L-T3 therapy 43%, desiccated thyroid 50%, other drug 7%, both L-T3 and desiccated 1%
Dose adjustments	Physician (blood samples) 44%, physician (symptoms) 17%, myself (symptoms) 28%, no answer (11%)
Duration T4 + T3 therapy	3–6 months 56%, 6–12 months 16%, 1–3 year 14%, >3 year 10%, no answer 4%
Most recent TSH	<0.01 mU/L 14%, 0.01–1.0 mU/L 54%, 1.0–2.5 mU/L 14%, 2.5–4.0 mU/L 8%, >4.0 mU/L 5%, no answer 4%
Response to T4 + T3 therapy	Miraculous 19%, much better 43%, better 22%, no difference 6%, worse 2%, no answer 8%

## What is the appropriate dosage of L-T4 + L-T3 combination therapy?

The recommended dosage of L-T4 + L-T3 combination therapy in the 2012 ETA guidelines is based on the assumption that euthyroidism can be reached simultaneously in all tissues of hypothyroid patients only by L-T4 + L-T3 in a dose ratio that mimics the physiological T4 to T3 secretion ratio by the human thyroid gland (which is close to 13:1 by weight) [1]. The assumption, derived from the experimental animal studies by Morreale de Escobar et al., may or may not be true. The pharmacodynamic equivalence of L-T4 and L-T3 has been assessed in a randomized, double-blind, cross-over study in 10 thyroidectomized patients [43]. The target (TSH ≥ 0.5 mU/L but ≥ 1.5 mU/L for at least 30 days) was reached by an average daily dose of either 115 ± 38.5 μg L-T4 or 40 ± 11 μg L-T3 (L-T4 to L-T3 ratio 0.36 ± 0.06). It was concluded that therapeutic substitution of L-T4 by L-T3 was achieved at a ratio of approximately 3:1. In other words, 30 μg T4 can be substituted by 10 μg T3 without changes in serum TSH. Thus, a simple method to arrive at the desired dose ratio between L-T4 and L-T3 is as follows. Dose *x* is the daily L-T4 dose in μg that has resulted in a normal serum TSH. The required daily L-T3 dose in μg (called *y*) is given by *y=x:20*. The requested daily L-T4 dose in μg (called *z*) is given by *z=x−3y* (Table 3) [1]. Whereas L-T4 can be given once daily, the daily L-T3 dose should be divided –if possible- in two doses, one before breakfast and the largest one before sleeping [1]. The rationale for splitting the daily L-T3 dose in two (or even three) gifts is the relatively short half-life of L-T3, peak serum T3 values at 2–4 h after ingestion, and a physiological diurnal variation in serum T3 with zenith around 4 am and nadir between 3 and 5 pm [44]. It is suggested to start T4 + T3 combination therapy in a L-T4:L-T3 dose ratio between 13:1 and 20:1 by weight [1]. The serum FT3/FT4 ratio (pmol/l to pmol/l) in hypothyroid patients replaced with L-T4 is 0.24 (IQR 0.20–0.28), lower than the value of 0.32 (IQR 0.27–0.37) in euthyroid controls [16]. In RCTs the serum FT3/FT4 ratio is 0.30 (IQR 0.25–0.45) during L-T4 + L-T3 combination therapy, higher than the value of 0.24 (IQR 0.18–0.25) during L-T4 monotherapy but still somewhat lower than in controls [1]. A retrospective observational study in Denmark reports on patients with persistent symptoms despite L-T4 therapy and normal serum TSH [45]. Treatment was changed into L-T4 + L-T3 combination therapy in a 17:1 ratio (weight/weight). After 12 months, 65% were responders and 35% nonresponders. There were no differences between both groups in the decrease of serum T4 (−18 and −4.5 nmol/L respectively) nor in the increase of serum T3 (+0.28 and +0.25 nmol/l, respectively).

**Table 3:** Simple method for calculating appropriate L-T4 and L-T3 dosages for T4 + T3 combination therapy [1]

T4 monotherapy dose x = L-T4 dose that normalized TSH	100 μg L-T4 = dose x	150 μg L-T4 = dose x	200 μg L-T4 = dose x
*T4 (dose z)+T3 (dose y) combination therapy*			
L-T3 dose *y* = *x*: 20 L-T4 dose *z* = *x*−3*y* L-T4 dose (round off) L-T4: L-T3 dose ratio	5 μg 85 μg 87.5 μg 17:1	7.5 μg 127.5 μg 125 μg 17:1	10 μg 170 μg 175 μg 17: 1

## Which preparations can be used in L-T4 + L-T3 combination therapy and how should their use be monitored?

The 2012 ETA guidelines recommend to use separate L-T4 and L-T3 tablets in combination therapy, as available combination tablets contain a L-T4/L-T3 dose ratio of 4:1, 5:1 and 10:1 (Table 4). These ratios are significantly different from the recommended ratios of 13:1 to 20:1. If dose adjustments are necessary, it is also more convenient to change the dose of just one of the components, preferably of L-T3 [1]. In view of the pharmacokinetics of L-T3, a slow release preparation of L-T3 would be welcome, but that has not been realized. Thyromax, a L-T3 tablet made with microcrystalline cellulose and magnesium stearate, was hoped to have a sustained T3 release profile, but it had a serum T3 profile similar to Cytomel [46, 47]. A single dose of T3 sulfate provided steady-state serum T3 concentrations for 48 h, but nothing more has been heard about this interesting observation [48]. What has been accomplished since 2012, however, is the availability of L-T3 tablets of low strength (like 5 μg Cytomel). Splitting tablets in halves comes in handy if low doses of 2.5 or 7.5 μg L-T3 are required. This development undoubtedly has been driven by great interest in the combination therapy. On the negative side of this development one should note an unacceptable increase in the price of L-T3 tablets implemented by particular pharmaceutical companies. E.g. in the UK, the price of a single 20 μg generic L-T3 tablet increased suddenly from 0.16 to 9.22 £. The total monthly cost of L-T3 prescriptions for NHS England was 758975 £ in August 2013; the figure was increased by almost ten times to 7018679 £ by July 2018, despite fewer prescriptions [49]. This all resulted in widespread patient concern, media coverage, and to a motion in the House of Lords. The motion, moved by Lord Hunt of Kings Heath, reads “That this house regrets that the Branded Health Service Medicines (Costs) Regulations 2018 do not propose any action be taken in respect of the high cost charged by Concordia and other companies for the drug Liothyronine for the treatment of hypothyroidism, thereby depriving patients of the use of that essential drug” [50]. Lastly, DTE was preferred by patients over L-T4 in a RCT [51] and in a website-based survey [5]. DTE is not recommended in guidelines, but patients’ preference for the drug should be explored in more depth.

**Table 4:** Available formulations of L-T3 tablets, L-T3 + L-T4 combination tablets, and desiccated thyroid extract [44]

Brand name	T3 dose	T4 dose	Availability
L-T3 tablets			
Cytomel	5, 25, 50 μg		USA, Canada, Holland
Thybon	20, 100 μg		UK
Tertroxin	20 μg		Australia, South Africa
Liotyr	5 μg (soft gel)		Italy
L-T3 + L-T4 tablets			
Prothyroid	10 μg	100 μg	Germany
Novothyral	5, 15, 20 μg	25, 75, 100 μg	Europe
Thyreotom forte	10, 30 μg	40, 120 μg	Czech republic
Desiccated thyroid extract			
Nature thyroid per 65 mg grain	9 μg	38 μg	USA
Westhroid pure per 65 mg grain	9 μg	38 μg	USA
NP thyroid per 60 mg grain	9 μg	38 μg	USA
Thyroid (erfa) per 60 mg grain	8 μg	35 μg	Europe, Canada
Armour thyroid per 60 mg grain	9 μg	38 μg	USA

## What are areas for future research on this topic?

Since the ETA guidelines were published in 2012, many issues surrounding T4 + T3 combination therapy have become more clear. Much progress has been made in elucidating the putative role of SNPs in type 2 deiodinase (like Thr92Ala), but why some patients on L-T4 keep persistent symptoms despite a normal serum TSH, remains obscure. The controversial issue of combination therapy in my opinion can only be solved by doing many more clinical trials. It is a sobering thought that so far none of the studies suggested in the 2012 guidelines, have been realized (Table 5). This is even more worrisome because dissatisfaction with treatment outcomes is growing, and the clinical problem now has obtained political overtones. Clinical research becomes more difficult when politics are involved.

**Table 5:** Suggestions for future research made in the 2012 ETA guidelines on the use of L-T4 + L-T3 in the treatment of hypothyroidism [1]

1. Prospective studies in hypothyroid patients starting L-T4 therapy, comparing baseline characteristics between those who will and those who will not be satisfied with the outcome of L-T4 monotherapy.
2. Trials investigating the L-T4/L-T3 dose ratio that best approximates the serum FT4/FT3 concentration ratio in healthy subjects.
3. Randomized clinical trials comparing L-T4 + L-T3 combination therapy and L-T4 monotherapy in hypothyroid patients who have persistent symptoms and/or are carriers of polymorphisms in thyroid hormone transporters and deiodinases.
4. Studies with a slow-release preparation of L-T3.
5. Prospective studies assessing the long-term efficacy and safety of L-T4 + L-T3 combination therapy.

None of these suggestions have been realized seven years later in June 2019
